# Acknowledgement to Reviewers of *High-Throughput* in 2019

**DOI:** 10.3390/ht9010002

**Published:** 2020-01-16

**Authors:** High-Throughput Editorial Office

**Affiliations:** MDPI, St. Alban-Anlage 66, 4052 Basel, Switzerland

The editorial team greatly appreciates the reviewers who have dedicated their considerable time and expertise to the journal’s rigorous editorial process over the past 12 months, regardless of whether the papers are finally published or not. In 2019, a total of 18 papers were published in the journal, with a median time to first decision of 16.5 days and a median time from submission to publication of 48 days. The editors would like to express their sincere gratitude to the following reviewers for their generous contribution in 2019:
Abarca, CarlaJiang, Xiao-TaoAchim, CristinaJones, MelissaAli, AliJuárez-Maldonado, AntonioAlShamrani, NouraKananavičiūtė, RūtaAnaya-López, Jose LuisKarlsson, IsabellaAshour, Mohamed LotfyKato, RyujiBec, KrzysztofKhalilia, Walid MahmoudBhattacharya, AnannyaLeung, IvanhoeBobko, NaraLong, YichengBorges, Alisson CarraroLuo, PingpingBurgos-Aceves, Mario AlbertoMráz, JaroslavBussadori, Sandra KalilNagai, KouheiCaldeira, MichaelPham, Thang V.Carlsson, Henrik (Sweden)Philip, NebuCarlsson, Henrik (USA)Qualley, DominicCastiglia, MartaRezvani, EhsanChan, Ka LungRodríguez-Ruano, Sonia MaríaChan, WanRouleau, EtienneChen, SimingRóżycki, BartoszChen, XunSalawu, EmmanuelCieplik, FabianSantos, ClesivanColeman, JonathanSchmitz, KatjaDe Rond, TristanSchneiderhan-Marra, NicoleDemertzis, KonstantinosShin, Dong-SikDeshpande, GauraviSignorini, CinziaElaiw, Ahmed M.Smyth, Gordon K.Font, RafaelSzostak, RomanGallo, GiovanniTan, Bertrand Chin-MingGarner, AngieTsoukalas, DimitrisGuo, JingshuUchida, ShizukaHu, GangVan Campen, Julia C.Hu, QiwenVladimirov, Vladimir I.Huang, DongweiWrenger, SabineHuang, Ruo-PanXia, TianHuang, XiaohuZhang, JinyangHufert, Frank T.Zheng, Yongqi Ji, Shaofei


